# Variability of the Skin Temperature from Wrist-Worn Device for Definition of Novel Digital Biomarkers of Glycemia

**DOI:** 10.3390/s25134038

**Published:** 2025-06-28

**Authors:** Agnese Piersanti, Martina Littero, Libera Lucia Del Giudice, Ilaria Marcantoni, Laura Burattini, Andrea Tura, Micaela Morettini

**Affiliations:** 1 CNR Institute of Neuroscience, Corso Stati Uniti 4, 35127 Padova, Italy; agnese.piersanti@in.cnr.it (A.P.); andrea.tura@cnr.it (A.T.); 2 Department of Information Engineering, Università Politecnica delle Marche, Via Brecce Bianche 12, 60131 Ancona, Italy; s1101253@studenti.univpm.it (M.L.); l.l.delgiudice@pm.univpm.it (L.L.D.G.); i.marcantoni@staff.univpm.it (I.M.); l.burattini@univpm.it (L.B.)

**Keywords:** digital biomarker, skin temperature, non-invasive glycemia measurement, continuous glucose monitoring, biomedical signal processing, diabetes, wearables, artificial intelligence, hypoglycemia, time in tight range

## Abstract

This study exploited the skin temperature signal derived from a wrist-worn wearable device to define potential digital biomarkers for glycemia levels. Characterization of the skin temperature signal measured through the Empatica E4 device was obtained in 16 subjects (data taken from a dataset freely available on PhysioNet) by deriving standard metrics and a set of novel metrics describing both the current and the retrospective behavior of the signal. For each subject and for each metric, values that correspond to when glycemia was inside the tight range (70–140 mg/dL) were compared through the Wilcoxon rank-sum test against those above or below the range. For hypoglycemia characterization (below range), retrospective behavior of skin temperature described by the metric *CV_T SD_* (standard deviation of the series of coefficient of variation) proved to be the most effective both in daytime and nighttime (100% and 50% of the analyzed subjects, respectively). On the other side, for hyperglycemia characterization (above range), differences were observed between daytime and nighttime, with current behavior of skin temperature, described by *M2_T_* (deviation from the reference value of 32 °C), being the most informative during daytime, whereas retrospective behavior, described by *SD_T hhmm_* (standard deviation of the series of means), showed the highest effectiveness during nighttime. Proposed variability features outperformed standard metrics, and in future studies, their integration with other digital biomarkers of glycemia could improve the performance of applications devoted to non-invasive detection of glycemic events.

## 1. Introduction

Glycemia monitoring is essential for patients suffering from diabetes, especially those under insulin treatment [1]. The traditional standard method used to measure glycemia relies on self-monitoring of blood glucose (SMBG), which requires individuals to obtain a blood sample, typically via a fingerstick, and analyze it using a portable glucometer. While SMBG provides accurate point-in-time glucose measurements, it does not offer continuous insights into glucose fluctuations throughout the day. Nowadays, continuous glucose monitoring (CGM) devices are becoming increasingly widespread, monitoring interstitial glucose levels every 1–5 min through a subcutaneous sensor that can be worn in everyday life for 7–14 days [2]. Beyond insulin treatment and diabetes management, a consumer-level CGM device (Lingo, Abbott, Abbott Park, IL, USA) has recently been released on the market for use by the general population to monitor glucose fluctuations in relation to lifestyle. This shift extends the interest in CGM use beyond clinical settings, fostering its potential for prevention in populations at risk of diabetes development, such as individuals with prediabetes, where early detection of impaired glucose tolerance patterns could inform timely interventions [3,4,5]. However, although CGM devices allow a minimally invasive measure, a fully non-invasive solution to monitor glycemia is still not available but desirable [6]. A number of different approaches are currently being explored for non-invasive glycemia assessment, such as iontophoretic extraction of glucose through the skin, Raman spectroscopy in aqueous humor, visible or near-infrared (NIR) spectroscopy, or fluorescence methods, among others, as reported by several review studies in the field [6,7,8,9,10]. However, these approaches face issues due to significant hardware and software challenges in meeting regulatory standards, which at present refer to those established for glucometers and CGMs [11,12], particularly regarding: (i) accuracy, typically assessed through Mean Absolute Relative Difference (MARD) and error grids, and (ii) long-term stability, which is often hindered by calibration and material degradation issues [13,14]. Such standards are even more difficult to meet for non-invasive prototypes since they do not involve direct blood or interstitial fluid glucose measurement and may be subject to external factors, such as skin thickness, that can introduce additional complexity and reduce accuracy.

A possible solution of interest is related to the field of “digital biomarkers” intended as digitally collected data from wearable devices that may be used as indicators of health outcomes [15,16]. While the correlation between these biomarkers and blood glucose levels alone may not be strong enough to serve as a direct surrogate measurement, they hold potential when integrated with non-invasive measurement tools to enhance accuracy. In fact, the discovery of digital biomarkers for glycemia or glycemic control from non-invasive devices (e.g., smartphones, smartwatches, or smart bands) in conjunction with artificial intelligence (AI) solutions has shown promising results in facilitating a non-invasive glycemia assessment [17,18,19,20,21,22]. As recently reviewed, data of interest for digital biomarker discovery in diabetes-related applications may come from electrocardiography, photoplethysmography, accelerometry, electrodermal activity, and skin temperature [23]. Some studies combined information from CGM with that of accelerometric signals from wearable sensors, enhancing CGM information for prediabetes or diabetes diagnosis [24,25,26]; other studies leveraged an open toolkit, namely FLIRT, for feature extraction from wearable sensors to detect hypoglycemia from completely non-invasive sensor-based features, among which are heart rate, heart rate variability, and tonic component of electrodermal activity [27,28,29]. In addition, other digital platforms are dedicated to digital biomarker discovery, integrating multi-sensor data, providing automated feature extraction—including CGM feature extraction modules—and the possibility to apply machine learning techniques to uncover meaningful physiological relationships [16].

Among the various physiological signals, skin temperature has been the least investigated one, although it is easily measurable and although it showed evidence of various physiological links to glycemic variations [30,31,32]. For instance, during hypoglycemia, a reduction in skin temperature can be observed, primarily driven by sweat evaporation and by the role of sympathetic nervous activity and circulating catecholamines in reducing the cutaneous blood flow, thus inducing vasoconstriction in skin vessels. During the postprandial phase, a drop in distal skin temperature is observed as blood flow shifts toward the gastrointestinal tract, and as the digestive process progresses, a compensatory vasodilation occurs, allowing nutrients to be transported peripherally. However, these responses are not uniform across individuals and conditions, and the underlying mechanisms remain not fully elucidated. Previous studies have primarily focused on skin temperature variations for detecting complications such as diabetic ulcers and infections and suggested the study of such variation in combination with electrodermal activity [33,34]. However, to the best of our knowledge, no study provided information on the engineered skin temperature features that may be used as digital biomarkers for glycemia. Thus, the aim of this study was to overcome this limitation by exploiting skin temperature signals derived from wearable devices to define potential digital biomarkers of glycemia.

## 2. Materials and Methods

### 2.1. Dataset Description

Data used in this study were retrieved from the BIG IDEAs Lab Glycemic Variability and Wearable Device Data (version 1.1.2), freely available on PhysioNet repository [35,36]. The study participants (P = 16) were subjects showing the presence of elevated blood glucose levels but still within the normal or prediabetic range (Hba1c < 5.7% or 5.7% ≤ Hba1c < 6.5%, respectively), thus not having diabetes. They were monitored for 8–10 days with i) a CGM minimally invasive device (the Dexcom G6, Dexcom, Inc., San Diego, CA, USA), which measures interstitial glucose every 5 min through a subcutaneous thin needle-type sensor, and with ii) a wrist-worn multi-sensor wearable factory-calibrated device (the Empatica E4, Empatica, Inc., Cambridge, MA, USA), which provides, among other measures, the skin temperature readings with a sampling frequency of 4 Hz. All the analyzed data were collected following the principles of the Declaration of Helsinki, under the approval of the local ethical authority and with all participants providing signed informed consent [20]. CGM interstitial glucose concentrations and skin temperature data were retrieved from each participant data folder (Dexcom.csv and TEMP.csv files, respectively). Each single file contained a two-column frame format with the timestamp in the first column and the related measured values in the second—in mg/dL for the CGM and in °C for the skin temperature. Study dataset characteristics are summarized in Table 1.

### 2.2. Preprocessing

Subjects with a percentage of missing CGM samples higher than 20% were removed from the analysis. To temporally align the skin temperature signal (sampled at 4 Hz) with respect to the time samples of the CGM signal (1 sample every 5 min), the former was windowed considering 5 min length windows, backward with respect to each CGM time sample. If glucose value was missing in a specific CGM time sample, the related temperature window was discarded. Then, each temperature window was screened for missing values; if occur, the temperature window was discarded together with the related CGM sample. Since, for each subject, the portion of the trace eligible for possible imputation, i.e., short gaps of less than 30 consecutive min, accounted for less than 0.5% of the entire trace (mean ± standard deviation across subjects: 0.45% ± 0.60%), the use of data imputation techniques was deemed not necessary and therefore was not performed to avoid introducing biases. Each temperature window was also screened to detect artifacts and/or outliers, and if present, the whole window was discarded. Artifacts were detected if temperature values lower than 28 °C were found. The threshold of 28 °C was selected by visually inspecting the data and considering that the data were recorded in real-life conditions, thus reaching lower values with respect to data recorded exclusively at room temperature, which usually are higher than 30 °C [37]; after computing mean and standard deviation for each window, temperature values outside the range mean ± 5 standard deviation were considered as outliers. Of note, outlier and artifact detection was carried out, ensuring that if at least one of the two conditions (i.e., presence of artifact or presence of outlier) is verified, the window is discarded. After the preprocessing steps, the ith window retained for the analysis was characterized by the skin temperature values *st_i1_*,…, *st_iN_* with N = 5 × 60 × 4 = 1200.

### 2.3. Analysis of Skin Temperature Signal: Standard Metrics and Definition of Novel Variability Metrics

Skin temperature signal was analyzed by computing a set of standard and novel variability metrics for each ith window. Computed metrics accounted for current and retrospective behavior of the signal. Metrics accounting for the current behavior were computed using only values inside the ith window; metrics accounting for the retrospective behavior were computed using values inside the ith window and all the previous windows. In the metric definition, T subscript is used to indicate that the metric refers to temperature.

#### 2.3.1. Metrics for the Current Behavior

For the ith window, the set of computed metrics for the current skin temperature behavior included basic statistical metrics like mean (*mean_Ti_*), median (*median_Ti_*), standard deviation (*SD_Ti_*), interquartile range (*IQR_Ti_*), and coefficient of variation (*CV_Ti_*), as well as mean amplitude deviation (*MAD_Ti_*), which is defined as

(1) $${MAD}_{Ti}=1.4826\cdot \sum_{k=1}^{N}\frac{\left|{st}_{ik}-{median}_{Ti}\right|}{N}$$

where the constant 1.4826 is a scale factor used to ensure asymptotically normal consistency [38].

Additionally, some novel metrics were defined, borrowing and adapting metrics commonly applied to the CGM domain [39]. Starting from the definition of the J-index given in the glycemic domain [40], an analogous mathematical index was defined for the skin temperature signal (*J_Ti_*). The index takes into account two components, namely the mean level and the variability (assessed through the standard deviation), according to the following formula:

(2) $$J_{Ti}=0.001\cdot {\left({mean}_{Ti}+{SD}_{Ti}\right)}^{2}$$

By making reference to the M-value index defined for the glycemic domain [41], two additional indexes (*M1_Ti_* e *M2_Ti_*) were proposed that quantify the deviation from a given reference value:

(3) $${M1}_{Ti}=\sum_{k=1}^{N}\frac{\left|{{10\cdot log(st}_{ik}/r_{1})}^{3}\right|}{N}$$

(4) $${M2}_{Ti}=\sum_{k=1}^{N}\frac{\left|{{10\cdot log(st}_{ik}/r_{2})}^{3}\right|}{N}$$

where *r*_1_ and *r*_2_ were set to 36 °C and 32 °C, respectively, to account for maximal and mean temperature reference values.

#### 2.3.2. Metrics for the Retrospective Behavior

For the ith window, two novel metrics based on retrospective coefficient of variation values were computed, considering the mean and the standard deviation of all coefficient of variation values related to the current and previous windows, respectively:

(5) $${CV}_{Ti\;mean}=\sum_{k=1}^{i}\frac{{CV}_{Tk}}{i}$$

(6) $${CV}_{Ti\;SD}=\frac{\sum_{k=1}^{i}{\left({CV}_{Tk}-{CV}_{Ti\;mean}\right)}^{2}}{i}$$

One additional metric was computed based on the computation of standard deviation of the retrospective mean values according to the following formula:

(7) $${SD}_{Ti\;hhmm}=\frac{\sum_{k=1}^{i}{\left({mean}_{Tk}-m\right)}^{2}}{i}$$

where m is the mean of all the temperature values across the ith and previous windows.

### 2.4. Stratification for Different Glycemic Levels

The metrics computed in the temperature signal were stratified in relation to the corresponding glycemia values in the CGM data to evaluate differences in normoglycemic vs. hyper/hypoglycemic conditions. Specifically, the metrics in ith window were classified according to the following criteria: (i) in range (IR) for CGM values in the interval 70–140 mg/dL, (ii) above range (AR) for CGM values higher than 140 mg/dL, or (iii) below range (BR) for CGM values lower than 70 mg/dL.

### 2.5. Statistical Analysis

Normality of distributions of the computed metrics was tested through Lilliefors test. A first exploratory analysis was conducted for each subject and for each temperature metric; values assumed by the metric when glycemia was AR or BR were compared with values assumed by the metric when glycemia was IR through the two-sided Wilcoxon rank sum test for equal medians. *p*-values were corrected for multiple testing using the Benjamini–Hochberg False Discovery Rate (FDR) method.

In addition, in order to evaluate whether the effect of the glycemic level on skin temperature metrics can be generalized beyond subject level, a linear mixed-effects model (LME) was employed, which includes glycemic level as a fixed effect and subject’s identifier as a random intercept. This approach accounts for repeated measurements within individuals and accommodates the unbalanced structure of the dataset. Separate models were fitted for comparisons between glycemic levels (i.e., AR vs. IR and BR vs. IR). In case of skewed distributions, LME was applied to square-root-transformed values, which can be preferred when variance is proportional to the mean [42]. *p*-values associated with the fixed effect of glycemic group were extracted and corrected for multiple comparisons across the computed metrics using FDR correction.

Comparison was conducted separately for daytime (from 6:00 to 21:00) and nighttime (from 21:00 to 6:00) periods, which were set based on visual inspection (Figure S1 in the Supplementary Material) and in agreement with the review by Vellei et al. [43]. Statistical significance was determined with a threshold of *p* < 0.05. Data are reported as median [25th percentile; 75th percentile] unless otherwise specified.

## 3. Results

**Figure 1:**
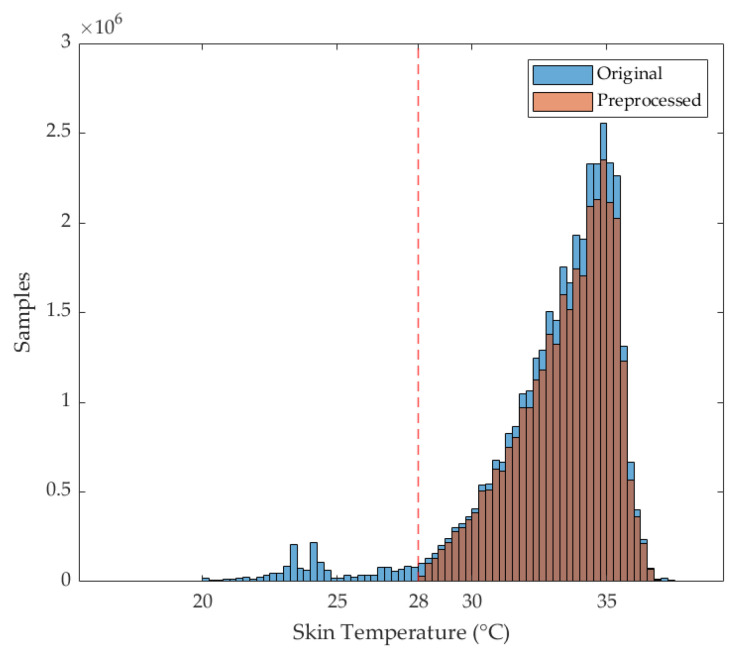
Histogram comparison of skin temperature distributions before and after preprocessing. The blue histogram represents the temperature values before preprocessing, while the orange histogram corresponds to skin temperature values after preprocessing (artifacts and outlier removal). Both histograms use a bin width of 0.25 °C. The x-axis indicates skin temperature in degrees Celsius (°C), and the y-axis represents the number of samples in each bin. The red dashed threshold is the cut-off set to 28 °C.

A total of 15 subjects out of 16 were included in the analysis. Indeed, subject 15 was excluded since more than 20% of CGM values were missing. The total number of windows before and after preprocessing was 34,647 and 26,987, respectively, with a mean ± standard deviation across subjects of 2310 ± 294 before and 1799 ± 290 after preprocessing, respectively. In terms of percentages, the mean ± standard deviation percentage of retained windows across subjects was 78% ± 11%. Distributions before and after preprocessing are shown in Figure 1. For all studied subjects, AR level (hyperglycemia) was always observed, both in daytime and nighttime. Metrics in the BR level (hypoglycemia) were computed for 6 out of 15 subjects, corresponding to those for whom hypoglycemia was observed. The group of subjects experiencing hypoglycemia partially differed between daytime and nighttime (P1, P3, P7, P10 vs. P1, P7, P8, P16, respectively).

**Figure 2:**
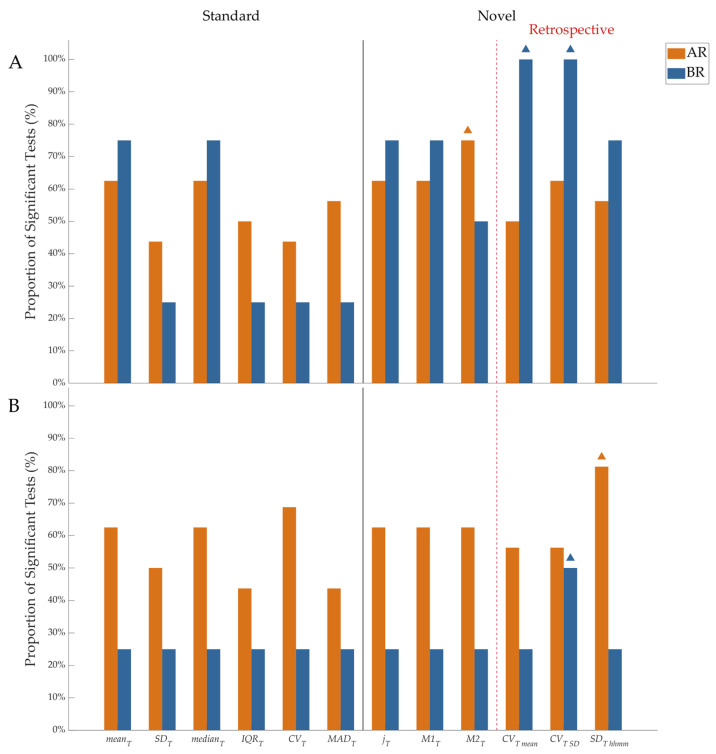
Performance of the temperature metrics in terms of their ability to detect statistically significant differences between AR/BR (hyperglycemia/hypoglycemia) conditions and IR condition for daytime (**A**) and for nighttime (**B**); orange triangle: best-performing metric for hyperglycemia; blue triangle: best-performing metric for hypoglycemia. The proportion of significant tests is expressed in terms of the percentage of subjects.

**Figure 3:**
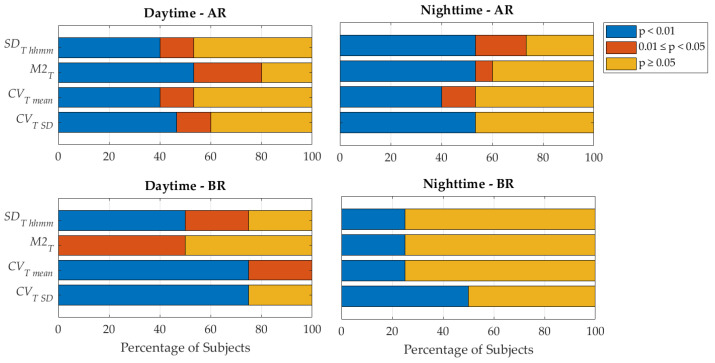
Performance of the four best-performing skin temperature metrics in terms of their ability to detect moderate (0.01 ≤ *p* < 0.05) and strong (*p* < 0.01) statistically significant differences between AR/BR (hyperglycemia/hypoglycemia) conditions and IR (in range) conditions for daytime (**right**) and for nighttime (**left**); the proportion of significant tests is expressed in terms of the percentage of subjects. Benjamini–Hochberg False Discovery Rate (FDR) correction is used.

The performance of the skin temperature metrics, in terms of their ability to detect statistically significant differences between AR/BR conditions and IR conditions, was reported in Figure 2. Following the Lilliefors test, all metrics computed in the IR condition violated the hypothesis of normality (see Figure S2 in Supplementary Material); thus, non-parametric tests were used in the following statistical analysis. The only exception was for LME models, for which values were corrected through square-root transformation, as the majority of the analyzed metrics showed a variance proportional to the mean (see example in Figure S3 in Supplementary Material) [42]. For daytime hypoglycemia detection (BR), only the retrospective metrics *CV_T mean_* and *CV_T SD_* reached statistical significance in 100% of subjects experiencing such hypoglycemic events (P1, P3, P7, P10), as shown in Figure 2A. For nighttime hypoglycemia detection (BR), *CV_T SD_* was the only metric showing differences for 50% of the four subjects experiencing hypoglycemic events (P8, P16), as shown in Figure 2B. For daytime hyperglycemia detection (AR), the *M2_T_* metric showed the best performances, whereas for nighttime hyperglycemia detection, the best performance was shown by *SD_T hhmm_*. As regards the best-performing metric for each case (*M2_T_*, *CV_T mean_*, *CV_T SD_*, and *SD_T hhmm_*), values for each subject were reported in Table 2, Table 3, Table 4 and Table 5, respectively. In Figure 3, each of the four best-performing temperature metrics ability to unveil moderate (0.01 ≤ *p* < 0.05) and strong (*p* < 0.01) statistically significant differences between AR/BR and IR is shown. Linear mixed-effects (LME) modeling revealed a statistically significant effect of glycemic levels on the evaluated skin temperature metrics after accounting for between-subject variability through random intercepts (for daytime hypoglycemia *M2_T_*, *CV_T mean_*, *CV_T SD_*, corrected *p* < 0.001; for nighttime hypoglycemia *M2_T_*, *CV_T mean_*, *CV_T SD_*, and *SD_T hhmm_*, corrected *p* < 0.03; for daytime hyperglycemia *SD_T hhmm_*, corrected *p* < 0.02; for nighttime hyperglycemia *M2_T_*, *CV_T mean_*, *CV_T SD_*, and *SD_T hhmm_*, corrected *p* < 0.001). This suggests that, overall, skin temperature metrics vary across glycemic levels.

## 4. Discussion

### 4.1. Novelty and Relevance

This study presents an in-depth characterization of the skin temperature signal measured through a wearable device by introducing novel metrics to analyze its variability in relation to different glycemic ranges (normal, hypoglycemia, and hyperglycemia) and comparing them with standard statistical metrics. The novel skin temperature metrics here defined outperformed standard statistical metrics in both hypoglycemia and hyperglycemia conditions and for both daytime and nighttime, thus indicating that such standard metrics are only partially effective in capturing skin temperature changes with glycemic levels and, for this reason, not adequate to be used as digital biomarkers for glycemia.

At the subject level, the set of the novel best-performing metrics included metrics describing the current behavior (i.e., *M2_T_*) as well as metrics describing the retrospective behavior (i.e., *CV_T SD_*, *CV_T mean_*, *SD_T hhmm_*) of the skin temperature signal. For hypoglycemia characterization, the retrospective metric *CV_T SD_* proved to be the most effective both in daytime (100% in Figure 2A) and nighttime (50% in Figure 2B). On the other side, for hyperglycemia characterization, differences were observed between daytime and nighttime, with current behavior of skin temperature (described by the *M2_T_* metric) being more informative during the day, whereas retrospective behavior (*SD_T hhmm_* metric) was more informative during the night. When generalizing beyond the subject’s level, results showed that the same set of the novel best-performing metrics is suitable to characterize both hypoglycemia and hyperglycemia during nighttime, while for daytime, *SD_T hhmm_* is the most effective to characterize hypoglycemia and hyperglycemia; in parallel, also *CV_T mean_* and *M2_T_* were effective in hypoglycemia characterization during daytime. In summary, the results obtained reinforce that skin temperature is a biosignal worth investigating in the field of digital biomarkers for glycemia and also extend the knowledge of the information led by skin temperature variability. Indeed, the concept of variability is well consolidated for some biosignals (such as heart rate variability for electrocardiograms or glycemic variability for continuous glucose monitoring data, to mention some), and this study adds the skin temperature signal among those that could be characterized through variability.

It has to be recalled that the set of novel variability features for skin temperature here defined is not intended to be used per se to predict exact glycemia values, but this exploratory study, which provided for the first time a pipeline to process the skin-temperature signal, can serve to delineate a more reliable source of information (compared with the raw signal) for AI-based approaches that estimate glycemic levels, if not in terms of absolute values, at least in terms of risk of occurrence of undesired glycemic events (hyperglycemia and, especially, hypoglycemia) [21,23,32,44,45,46,47]. Indeed, the use of such approaches benefits from being exposed to a high number of features, and this set of temperature metrics can complement information either from digital biomarkers derived from other signals (electrocardiography, photoplethysmography, accelerometry, electrodermal activity, among others), similarly measured through wearable devices, or from non-invasive instruments that provide direct measures (such as optical sensors, near-infrared spectroscopy, Raman spectroscopy, or electromagnetic sensing). This complementarity may help to address one of the key challenges that the current technologies for non-invasive glycemia assessment face in reaching market-ready accuracy.

### 4.2. Advantages and Comments on the Methodology

This study presents a series of advantages. One of the advantages relies on the separate analysis performed for daytime and nighttime. Overall data quality is higher during nighttime compared with daytime, lowering the possibility of movement artifacts or disconnections. Moreover, skin temperature during the day may be heavily affected by changes induced by ambient temperature [48]. We could not investigate in detail the influence of ambient temperature since this information was not reported in the dataset. Nevertheless, separating the analysis for daytime and nighttime limited the effect of this confounding factor, and the proposed metrics showed satisfying results for both daytime and nighttime, thus enforcing their suitability in capturing glycemic changes. A second advantage can be found in the accurate preprocessing of the skin temperature signal devoted to the artifact and/or outlier detection and removal. Indeed, differently from other biological signals, to date, no reference procedure exists for processing the skin temperature signal measured from wearable devices, and hence, data quality is often disregarded. Based on the description of the most common artifacts given in the study by Böttcher et al. [49], the present study implemented for the first time a pipeline that can be used as a reference for other studies in which skin temperature signals are measured from wearable devices. Regarding the criterion we applied to identify artifacts in the skin temperature signal, we decided on a lower threshold of 28 °C because we assessed it to be the appropriate one when recordings are obtained under real-life conditions and not only at room temperature. Notably, in the preprocessing phase, we made the choice not to use data imputation methodologies since the presence of short (imputable) gaps was not prevalent in the dataset; however, for a more comprehensive preprocessing pipeline for the skin temperature signal, we recommend assessing data quality and leveraging imputation techniques such as spline and polynomial-based interpolation, already used for the CGM signal, and that can be readily used also for skin temperature, which shares similar slow time dynamics [50,51]. As regards the minimum requirement for data loss of the skin temperature signal to be considered acceptable, for this study, we have borrowed the common minimum percentage of data that is recommended for the CGM signal in the consensus for a time in the range [52,53], which is equal to at least 70% of available data. Eventually, the definition of the glycemic intervals represents an additional advantage of this study; indeed, to set the glycemic cut-offs, this study relied on the definition of a recently introduced blood glucose management indicator called Time in Tight Range (TITR, being the time of glycemia within the 70–140 mg/dL interval), which represents a more “strict” metric for achieving glucose normalization compared with the Time in Range (TIR, time within the 70–180 mg/dL interval) [54]. Because the study population is at risk of developing diabetes but has not yet progressed to overt disease, setting such strict glycemic cut-offs is, in fact, particularly important to increase the chance of identifying subtle glucose dysregulations that may precede disease onset.

Although the diffusion of CGM devices has increased the amount of collected data, freely available CGM datasets are still rare, and those with the simultaneous acquisition of skin temperature data are even less so. As far as we know, two other available CGM datasets that also measured skin temperature exist: (i) the D1NAMO dataset by Doubusson et al. [55], measuring proximal skin temperature through Zephir Bioharness 3 (Zephyr Technology, Annapolis, MD, USA), and (ii) the Ohio T1DM dataset by Marling and Bunescu [56], measuring skin temperature using two different wrist-worn devices, with six subjects wearing the Basis Peak and the other six subjects wearing the Empatica Embrace (Empatica, Inc., Cambridge, MA, USA). We excluded such datasets to avoid confounding effects due to differences in thermoregulatory responses between distal and proximal skin regions, as well as differences in the device used; moreover, we also decided to exclude such datasets since they pertain to subjects with type 1 diabetes for whom exogenous insulin administration could further influence thermoregulatory responses, thus complicating the interpretation. Instead, the dataset considered in the present study has the advantage of ensuring consistency and comparability in distal skin temperature measurements without confounding effects introduced by the device and/or exogenous insulin administration. In addition to its multimodal nature, useful for further analysis, this dataset showed good data completeness, and, being collected in free-living conditions, it resembles real-world settings. Of note, in previous studies using the same dataset, the skin temperature signal was used as one of the non-invasive data sources to estimate blood glucose levels [26,57,58]. However, in all cases, either the raw signal was used as input for deep learning algorithms, or the features extracted from the skin temperature signal were limited to standard statistical features (e.g., mean, standard deviation, 1st quartile) without exploring a more advanced characterization of the signal. Despite this, the use of the skin temperature signal has proven effective when combined with other signals like photoplethysmography and electrodermal activity to predict glucose levels, demonstrating performance improvements with respect to the use of a single signal as a biomarker, thus further confirming the thesis that a multimodal approach would be valuable.

### 4.3. Limitations and Future Work

A significant limitation of this study is that several known confounders of skin temperature were not measured or controlled. In particular, the population under investigation limits the understanding of how individual variability affects the results. Indeed, individuals’ baseline core temperature is known to show meaningful variation due to many factors like demographic characteristics (e.g., age, sex, and BMI), presence of comorbidities and physiology, but also in relation to other factors that are unknown [59]. This is even more challenging when temperature is recorded from the skin and hence does not reflect the core body temperature exclusively but also changes induced by physical activity, emotions, hormonal levels, humidity, ambient temperature, or other environmental factors such as temperature variations influenced by electromagnetic fields [60,61]. However, we attempted to mitigate their influence by (i) focusing on glycemic risk categories rather than exact glucose values, (ii) analyzing nighttime and daytime data separately to account for circadian variability, and (iii) performing exploratory subject-specific analyses to reduce behavioral variability and inter-individual noise. Despite these precautions, the omission of key confounders, sensitivity analyses, and external validation mandate that the findings reported here should be interpreted with caution. Thus, the potential influence of unmeasured confounding factors cannot be fully excluded and should be addressed in future studies, where future protocols should include ad hoc acquisitions.

Another limitation lies in the characteristics of the study population, which limits results generalizability to datasets including subjects with different characteristics (e.g., insulin-treated diabetes). This is particularly evident when considering the hypoglycemic condition since the evaluation of the metrics performance relies on a subset of six out of fifteen subjects. Indeed, although hypoglycemic events are not common in general, this study included only subjects at risk of developing diabetes, who are less prone to experience hypoglycemic events than subjects with overt diabetes. Since the results obtained for hypoglycemic events are the most critical for real applications, a comprehensive evaluation of the ability of the proposed metrics to show variation with hypoglycemic events will require data with an adequate hypoglycemia occurrence and will be a matter of future investigation. Beyond the targeted population at risk of diabetes development, future studies in larger populations and in different types of diabetes, such as type 1, type 2, and gestational diabetes, could investigate the ability of the proposed metrics to capture such variability among different individuals having different conditions.

Moreover, since results showed that not all the skin temperature metrics can be generalized for all the tested conditions, caution will be needed when training models. This finding suggests the importance of exploring personalized detection models to account for individual variability. Considering intra-day variability, the separate analysis of daytime and nighttime was also important to account for circadian rhythm effects on skin temperature. Indeed, there is evidence that at sleep onset, an increase in distal skin temperature occurs due to a higher blood flow to the extremities [62,63]. The choice of using a standard period to define daytime and nighttime as a proxy of sleep-awake periods was driven by the fact that although the Empatica E4 device provides actigraphy data related to the subject’s actual sleep periods, such data were unfortunately not available in the dataset analyzed in this study. Thus, we have relied on visual inspection, which provided results similar to those observed in the study by Vellei et al. [43]. Nowadays, however, different sleep/wake detection algorithms have been developed starting from accelerometric data [64]; thus, implementing one of these algorithms could allow for subject-specific sleep/wake period definitions, which could be explored in future studies.

As pointed out, future research should also explore the use of a pool of non-invasive digital biomarkers of glycemia, including the skin temperature metrics defined in this study, to develop machine learning-based approaches for the non-invasive detection of hyperglycemic and hypoglycemic events. In the latter context, room for optimization will be possible by investigating how the variability of the skin temperature relates to glycemic variability, whose features can be assessed through a series of software packages and tools already available and tested by our group for reliability [65].

## 5. Conclusions

In conclusion, the present study proposed a set of metrics that can be extracted from the skin temperature signal measured by a wrist-worn device and whose variation is informative about the occurrence of hypoglycemic/hyperglycemic events. These metrics, describing skin-temperature variability for the first time, can act as digital biomarkers and may be exploited as features in place of the raw signal to improve applications devoted to non-invasive glycemia assessment. Moreover, given that such metrics were designed and tested on individuals at risk of developing diabetes, who are characterized by the occurrence of hyperglycemia rather than hypoglycemia, the potential of such metrics at present is higher in applications focused on complementing non-invasive glucose monitoring systems to be used as screening tools for pre-diabetes.

## Figures and Tables

**Table 1:** Summary including participants demographics and sensors measurements characteristics.

Participants	
P	16
Sex (M/F)	7M/9F
Age (years)	NR (inclusion criteria 35–65 years)
Hba1c (%)	5.7 ± 0.3
Monitoring period	8–10 days
**CGM data**	
Total number of samples	36,872
Number of samples across subjects	2318 ± 302
Percent of data completeness across subjects (%)	97 ± 6
**Skin temperature data**	
Total number of samples	37,543,280
Number of samples across subjects	2,346,455 ± 400,741
Percent of data completeness across subjects (%)	79 ± 19

Characteristics are expressed as mean ± standard deviation, where applicable. Hba1c: glycated hemoglobin; CGM: continuous glucose monitoring; P: number of participants; M: male; F: female; NR: not reported. Bold labels identify different sections of the table.

**Table 2:** ${M2}_{T}$·10^−2^ (unitless) values for glycemia classified as in range (IR), above range (AR) or below range (BR) for all subjects during daytime and nighttime.

Subject	Daytime			Nighttime		
	IR	AR	BR	IR	AR	BR
S1	1.07 [0.21; 4.04]	**3.52** [0.93; 5.01] *	0.88 [0.53; 3.82]	12.86 [8.88; 15.11]	**6.35** [5.17; 13.12] *	11.59 [11.01; 12.17]
S2	3.30 [1.62; 5.03]	**3.72** [1.90; 5.99] *	-	3.52 [1.82; 5.12]	3.29 [1.75; 4.89]	-
S3	1.11 [0.16; 4.02]	**6.60** [1.88; 9.79] *	**0.04** [0.00; 0.11] *	3.73 [1.25; 7.57]	**8.88** [1.45; 13.18] *	-
S4	2.96 [0.57; 6.27]	**1.55** [0.10; 4.53] *	-	6.85 [3.55; 8.93]	6.72 [2.87; 9.29]	-
S5	0.36 [0.06; 1.38]	0.48 [0.00; 1.69]	-	1.68 [0.56; 3.76]	**10.08** [9.25; 10.92] *	-
S6	1.27 [0.20; 3.64]	**0.45** [0.13; 1.84] *	-	1.84 [0.56; 3.47]	**0.51** [0.12; 1.72] *	-
S7	0.57 [0.05; 3.88]	**2.49** [0.35; 7.90] *	**0.26** [0.03; 1.40] *	4.58 [1.13; 6.85]	**0.10** [0.00; 0.57] *	**0.01** [0.00; 0.08] *
S8	1.38 [0.26; 4.42]	**0.81** [0.10; 2.08] *	-	4.12 [0.91; 6.77]	0.28 [0.22; 0.33]	3.39 [1.66; 4.53]
S9	1.31 [0.14; 3.69]	**0.48** [0.04; 3.09] *	-	5.59 [3.26; 7.79]	**3.17** [1.51; 5.49] *	-
S10	0.84 [0.21; 2.41]	**0.42** [0.07; 1.64] *	0.62 [0.12; 2.76]	6.86 [4.43; 8.32]	**0.04** [0.01; 0.40] *	-
S11	0.47 [0.05; 1.96]	0.53 [0.07; 2.71]	-	7.59 [5.07; 9.03]	6.93 [5.29; 8.82]	-
S12	0.74 [0.09; 3.01]	**1.94** [0.43; 4.19] *	-	3.93 [1.14; 6.00]	**2.15** [0.84; 5.47] *	-
S13	0.73 [0.12; 2.48]	**0.14** [0.02; 0.94] *	-	1.61 [0.25; 3.67]	**0.21** [0.03; 0.94] *	-
S14	0.43 [0.05; 2.90]	**1.13** [0.33; 5.26] *	-	5.99 [3.60; 8.19]	4.53 [1.72; 8.29]	-
S16	1.52 [0.25; 5.20]	1.56 [0.21; 4.29]	-	4.17 [1.46; 6.49]	**7.39** **[4.56; 8.21]**	2.02 [1.64; 2.60]

Bold: statistically significant difference for comparison against in-range (IR) values assessed through a two-sided Wilcoxon rank sum test for equal medians (*p* < 0.05); * indicates Benjamini–Hochberg False Discovery Rate (FDR) corrected *p* < 0.05; - indicates that the subject did not show any glycemic value classified as below range (BR).

**Table 3:** ${CV}_{T\;mean}$ (unitless) values for glycemia classified as in range (IR), above range (AR) or below range (BR) for all subjects during daytime and nighttime.

Subject	Daytime			Nighttime		
	IR	AR	BR	IR	AR	BR
S1	0.35 [0.34; 0.36]	0.35 [0.35; 0.35]	**0.39** **[0.37; 0.40]** *	0.20 [0.20; 0.21]	**0.22** **[0.20; 0.22]** *	0.20 [0.20; 0.20]
S2	0.26 [0.25; 0.28]	**0.25** **[0.25; 0.27]** *	-	0.16 [0.16; 0.16]	**0.16** **[0.15; 0.16]** *	-
S3	0.57 [0.54; 0.60]	**0.51** **[0.50; 0.55]** *	**0.50** **[0.50; 0.50]** *	0.34 [0.33; 0.35]	**0.32** **[0.31; 0.33]** *	-
S4	0.38 [0.38; 0.40]	0.38 [0.38; 0.41]	-	0.19 [0.17; 0.23]	**0.23** **[0.17; 0.23]** *	-
S5	0.34 [0.33; 0.34]	**0.32** **[0.30; 0.33]** *	-	0.26 [0.26; 0.27]	**0.28** **[0.27; 0.28]** *	-
S6	0.36 [0.34; 0.38]	0.35 [0.35; 0.36]	-	0.26 [0.24; 0.27]	0.26 [0.24; 0.27]	-
S7	0.41 [0.39; 0.43]	**0.43** **[0.42; 0.44]** *	**0.44** **[0.41; 0.45]** *	0.29 [0.28; 0.31]	**0.31** **[0.30; 0.31]**	0.29 [0.28; 0.29]
S8	0.39 [0.39; 0.40]	**0.39** **[0.39; 0.39]** *	-	0.28 [0.26; 0.30]	0.30 [0.30; 0.30]	**0.41** **[0.36; 0.43]** *
S9	0.43 [0.40; 0.47]	0.42 [0.40; 0.46]	-	0.27 [0.23; 0.30]	**0.31** **[0.27; 0.41]** *	-
S10	0.47 [0.47; 0.48]	**0.47** **[0.46; 0.48]** *	**0.46** **[0.46; 0.47]** *	0.17 [0.16; 0.18]	0.17 [0.17; 0.17]	-
S11	0.41 [0.35; 0.42]	**0.42** **[0.36; 0.42]** *	-	0.12 [0.11; 0.12]	**0.12** **[0.11; 0.14]** *	-
S12	0.71 [0.70; 0.76]	0.71 [0.70; 0.75]	-	0.36 [0.35; 0.39]	**0.39** **[0.36; 0.40]** *	-
S13	0.35 [0.34; 0.36]	0.35 [0.34; 0.35]	-	0.25 [0.24; 0.26]	0.25 [0.24; 0.26]	-
S14	0.40 [0.34; 0.41]	**0.41** [0.40; 0.41]*	-	0.24 [0.23; 0.27]	0.24 [0.23; 0.24]	-
S16	0.47 [0.46; 0.51]	0.47 [0.45; 0.51]	-	0.28 [0.28; 0.29]	0.28 [0.27; 0.28]	0.28 [0.28; 0.28]

Bold: statistically significant difference for comparison against in-range (IR) values assessed through a two-sided Wilcoxon rank sum test for equal medians (*p* < 0.05); * indicates Benjamini–Hochberg False Discovery Rate (FDR) corrected *p* < 0.05; - indicates that the subject did not show any glycemic value classified as below range (BR).

**Table 4:** ${CV}_{T\;SD}$ (unitless) values for glycemia classified as in range (IR), above range (AR), or below range (BR) for all subjects during daytime and nighttime.

Subject	Daytime			Nighttime		
	IR	AR	BR	IR	AR	BR
S1	0.49 [0.47; 0.54]	**0.44** **[0.44; 0.47]** *	**0.19** **[0.17; 0.22]** *	0.47 [0.28; 0.51]	**0.50** **[0.49; 0.51]** *	0.28 [0.28; 0.29]
S2	0.23 [0.21; 0.26]	**0.22** **[0.20; 0.24]** *	-	0.16 [0.15; 0.18]	**0.15** **[0.10; 0.18]** *	-
S3	0.43 [0.40; 0.45]	**0.37** **[0.36; 0.42]** *	**0.36** **[0.36; 0.36]** *	0.34 [0.29; 0.35]	**0.27** **[0.27; 0.28]** *	-
S4	0.25 [0.25; 0.28]	0.25 [0.25; 0.29]	-	0.26 [0.19; 0.28]	**0.28** **[0.20; 0.28]** *	-
S5	0.22 [0.22; 0.23]	**0.22** **[0.21; 0.22]** *	-	0.20 [0.20; 0.21]	**0.23** **[0.23; 0.23]** *	-
S6	0.29 [0.27; 0.34]	**0.32** **[0.32; 0.33]** *	-	0.23 [0.20; 0.29]	0.23 [0.20; 0.27]	-
S7	0.38 [0.34; 0.39]	**0.39** **[0.39; 0.40]** *	**0.40** **[0.38; 0.40]** *	0.31 [0.29; 0.34]	0.32 [0.32; 0.32]	0.29 [0.29; 0.29]
S8	0.32 [0.32; 0.33]	**0.32** **[0.31; 0.32]** *	-	0.24 [0.24; 0.27]	0.26 [0.26; 0.26]	**0.34** **[0.32; 0.36]** *
S9	0.31 [0.29; 0.33]	**0.30** **[0.29; 0.32]**	-	0.29 [0.26; 0.33]	**0.34** **[0.30; 0.40]** *	-
S10	0.40 [0.36; 0.42]	0.40 [0.37; 0.41]	**0.36** **[0.36; 0.39]**	0.24 [0.23; 0.27]	0.24 [0.23; 0.28]	-
S11	0.33 [0.26; 0.36]	**0.36** **[0.27; 0.36]** *	-	0.13 [0.13; 0.14]	**0.14** **[0.12; 0.16]** *	-
S12	0.53 [0.51; 0.55]	0.53 [0.50; 0.55]	-	0.42 [0.41; 0.44]	**0.45** **[0.41; 0.46]** *	-
S13	0.28 [0.26; 0.28]	**0.28** **[0.26; 0.29]** *	-	0.23 [0.20; 0.24]	0.23 [0.20; 0.23]	-
S14	0.30 [0.25; 0.31]	0.30 [0.29; 0.31]	-	0.25 [0.25; 0.26]	0.26 [0.24; 0.27]	-
S16	0.40 [0.39; 0.43]	0.40 [0.39; 0.44]	-	0.30 [0.26; 0.30]	**0.27** **[0.27; 0.27]**	**0.32** **[0.32; 0.32]** *

Bold: statistically significant difference for comparison against in-range (IR) values assessed through a two-sided Wilcoxon rank sum test for equal medians (*p* < 0.05); * indicates Benjamini–Hochberg False Discovery Rate (FDR) corrected *p* < 0.05; - indicates that the subject did not show any glycemic value classified as below range (BR).

**Table 5:** ${SD}_{T\;hhmm}$ (°C) values for glycemia classified as in range (IR), above range (AR), or below range (BR) for all subjects during daytime and nighttime.

Subject	Daytime			Nighttime		
	IR	AR	BR	IR	AR	BR
S1	1.79 [1.72; 1.86]	1.76 [1.75; 1.80]	**0.28** **[0.22; 0.50]** *	0.77 [0.66; 0.84]	0.75 [0.66; 0.82]	0.78 [0.78; 0.78]
S2	1.21 [1.09; 1.31]	**1.15** **[1.08; 1.24]** *	-	0.65 [0.63; 0.67]	**0.64** **[0.49; 0.66]** *	-
S3	1.37 [1.23; 1.39]	**1.38** **[1.30; 1.44]** *	**1.40** **[1.40; 1.40]** *	0.98 [0.86; 1.58]	**0.80** **[0.79; 0.81]** *	-
S4	1.14 [1.12; 1.17]	1.14 [1.12; 1.16]	-	0.78 [0.76; 0.80]	**0.80** **[0.80; 0.77]** *	-
S5	0.92 [0.88; 0.93]	**0.88** **[0.71; 0.93]** *	-	0.86 [0.83; 0.89]	**0.77** **[0.76; 0.96]** *	-
S6	1.55 [1.47; 1.62]	1.54 [1.49; 1.55]	-	0.92 [0.89; 0.95]	**0.90** **[0.88; 0.27]** *	-
S7	2.05 [1.98; 2.22]	**1.97** **[1.96; 2.04]** *	**1.99** **[1.96; 2.07]** *	1.20 [1.17; 1.24]	**1.29** **[1.29; 1.30]** *	1.18 [1.17; 1.18]
S8	2.01 [1.95; 2.06]	**1.96** **[1.94; 2.01]** *	-	1.53 [1.47; 1.62]	**1.73** **[1.73; 1.74]**	**1.25** **[1.05; 1.25]** *
S9	1.46 [1.43; 1.50]	1.46 [1.45; 1.49]	-	1.16 [1.04; 1.22]	**1.27** **[1.18; 1.50]** *	-
S10	1.10 [1.06; 1.12]	**1.10** **[1.08; 1.14]** *	1.12 [1.09; 1.12]	0.83 [0.75; 0.87]	0.85 [0.81; 0.88]	-
S11	1.45 [1.14; 1.48]	**1.45** **[1.27; 1.49]** *	-	0.41 [0.34; 0.43]	**0.42** **[0.32; 0.45]** *	-
S12	1.68 [1.67; 1.71]	**1.71** **[1.67; 1.73]**	-	1.30 [1.22; 1.35]	**1.31** **[1.22; 1.32]** *	-
S13	1.23 [0.92; 1.26]	1.23 [1.19; 1.26]	-	1.24 [1.05; 1.25]	**1.25** **[1.11; 1.29]** *	-
S14	1.54 [1.46; 1.61]	**1.50** **[1.45; 1.59]** *	-	0.89 [0.85; 0.92]	**0.91** **[0.90; 0.92]**	-
S16	2.03 [1.96; 2.03]	2.04 [1.94; 2.06]	-	2.12 [2.02; 2.44]	**2.52** **[2.50; 2.53]** *	2.20 [2.19; 2.20]

Bold: statistically significant difference for comparison against in-range (IR) values assessed through a two-sided Wilcoxon rank sum test for equal medians (*p* < 0.05); * indicates Benjamini–Hochberg False Discovery Rate (FDR) corrected *p* < 0.05; - indicates that the subject did not show any glycemic value classified as below range (BR).

## Data Availability

The data used in this study belong to a database freely available in PhysioNet at https://physionet.org/content/big-ideas-glycemic-wearable/1.1.2/, accessed on 9 January 2024.

## Supplementary Materials

The following supporting information can be downloaded at: https://www.mdpi.com/article/10.3390/s25134038/s1, Figure S1: Overall temperature trend over the day; Figure S2: Normality test *p*-values; Figure S3: Example of Mean vs Variance graph.
