# Supplementary material for: Variability of the Skin Temperature from Wrist-Worn Device for Definition of Novel Digital Biomarkers of Glycemia
Source: Sensors (Basel). 2025 Jun 28;25(13):4038. doi: 10.3390/s25134038 (PMC12251718; doi:10.3390/s25134038)
Supplement: Supplementary file 1 [file sensors-25-04038-s001.zip › sensors-3639862-supplementary.pdf]

Supplementary Material

Supplementary Figures:

Figure S1..page 1

Figure S2..page 2

Figure S3..page 2

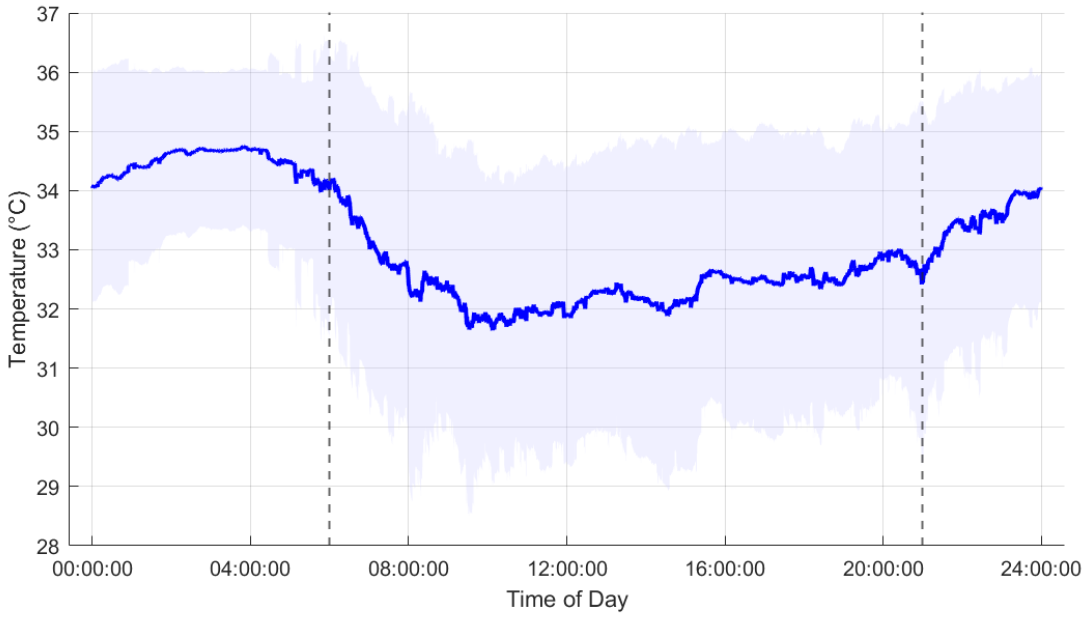

Figure S1: Overall temperature trend over the day (mean over subjects across different times of the day). The blue line represents the mean temperature and the shaded area corresponds to ±1 standard deviation. Changes in concavity (dashed vertical lines) occurred at 6:00:00 and at 21:00:00.

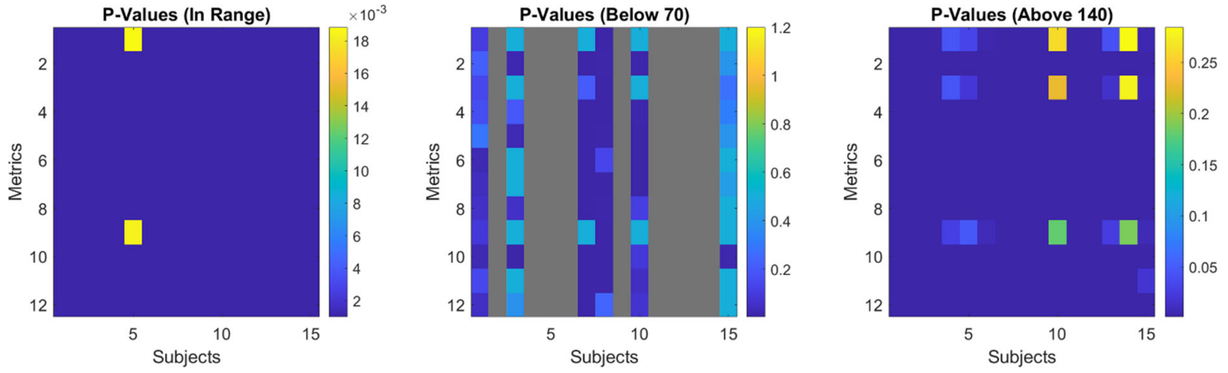

Figure S2: p-values obtained by the Lilliefors test to test normality for all the 12 skin temperature metrics computed from values corresponding to glucose levels inside, outside, below and above the target range. For glucose levels inside the target range (In Range), all metrics violated the hypothesis of normality (p<0.05). Note: gray color is used for subjects that did not show any hypoglycemic value (Below 70).

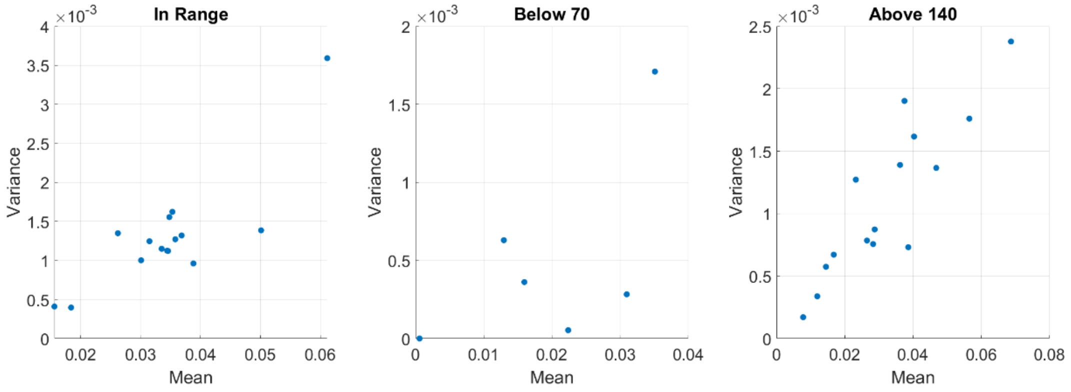

Figure S3: Example of Mean vs Variance graph obtained for the skin temperature metric $M2_T$ computed from values corresponding to glucose levels inside, below and above the target range. The graph show proportionality of Variance with respect to Mean.
